# Efficacy and safety of eravacycline versus tigecycline for complicated intra-abdominal infections in the ICU: a multicenter, single-blind, parallel randomized controlled trial study protocol

**DOI:** 10.3389/fmed.2024.1496402

**Published:** 2024-11-22

**Authors:** Jin Jie Liu, Dong Dong Guo, Meng Xing Wang, Yan Zhao Li, Hang Li, Si Bo Liu, Rong Li Yang, Dian Hong Zhang

**Affiliations:** ^1^Department of General Medicine, Central Hospital of Dalian University of Technology (Dalian Municipal Central Hospital), Dalian, China; ^2^Intensive Care Unit, Ansteel Group General Hospital, Anshan, China; ^3^China National Clinical Research Center for Neurological Diseases, Beijing, China; ^4^Department of Neurosurgery, Affiliated Zhongshan Hospital of Dalian University, Dalian, China; ^5^Department of Geriatrics, Affiliated Dalian Friendship Hospital of Dalian Medical University, Dalian, China; ^6^Intensive Care Unit, Central Hospital of Dalian University of Technology (Dalian Municipal Central Hospital), Dalian, China

**Keywords:** intraabdominal infections, eravacycline, tigecycline, intensive care unit, clinical trial protocol

## Abstract

**Background:**

Complicated intra-abdominal infections (cIAIs), often caused by multidrug-resistant bacteria such as carbapenem-resistant *A. baumannii* (CRAB) and carbapenem-resistant Enterobacteriaceae (CRE) are a critical challenge in ICUs. Owing to their high mortality and treatment failure rates, there is an urgent need for effective therapies. This trial will compare eravacycline to tigecycline for treating cIAIs in patients in the ICU, aiming to provide a superior treatment option.

**Methods:**

This is a multicenter, single-blind, parallel randomized controlled trial. Adult patients in the ICU with complex abdominal infections who meet the eligibility criteria will be included. The main outcome is the all-cause 30-day mortality of patients in clinically evaluable and microbiologically evaluable populations. Secondary outcomes include the proportion of total responsive patients in the clinically evaluable population at the end of treatment and test of cure visits; the proportion of total responsive patients in the microbiologically evaluable population at the end of treatment and test of cure visits; and ICU hospitalization time and costs. Safety assessments include the incidence of various adverse events and changes in clinical laboratory test results. The subjects will be randomly assigned to receive treatment with either eravacycline or tigecycline at a 1:1 ratio. The all-cause mortality rates of patients treated with eravacycline and TGC were 17.7 and 18.7%, respectively, with an estimated actual mortality rate of 0.95. A total sample size of 262 subjects is required to reach 80% power with an *α* of 0.05. Considering a 10% loss rate, 292 patients will be enrolled and randomly assigned to the three groups in equal proportions.

**Ethics and communication:**

This trial was approved by the Ethics Committee of Ansteel Group General Hospital. The communication plan includes presentations at scientific conferences, scientific publications, and presentations to the public through nonprofessional media.

**Clinical trial registration:**

https://clinicaltrials.gov/, ChiCTR2300078646.

## 1 Introduction

Complicated intra-abdominal infections (cIAIs) pose a significant challenge in the field of critical care medicine, particularly because of the involvement of multidrug-resistant (MDR) pathogens. Carbapenem-resistant organisms (CROs), a subset of MDR bacteria commonly associated with cIAIs, are increasingly recognized for their high incidence, strong resistance profiles, high treatment failure rates, and high mortality rates (1). These bacteria, including carbapenem-resistant *A. baumannii* (CRAB) and carbapenem-resistant Enterobacteriaceae (CRE), had alarming detection rates of 71.2–71.9% and 22.6–24.2%, respectively, in the 2022 CHINET China bacterial resistance monitoring report (2). These incidences highlight the severity of drug resistance issues in recent years.

The clinical outcomes associated with cIAIs are notably poor, often leading to increased ICU occupancy rates, prolonged hospital stays, and a significantly increased mortality rate (3–5). The economic burden of such infections is substantial, with CRE alone causing annual losses of $1.4 billion to hospitals, $800 million to third-party payers, and $2.8 billion to society in the United States (6). Given the profound impact of cIAIs on patient prognosis and healthcare systems, there is an urgent need for effective treatment strategies.

The current therapeutic landscape for MDR bacterial infections, including those complicated with cIAIs, is limited and inadequate. The high initial treatment failure rate of 81.3% for MDR bacterial infections underscores the urgency for new treatment options (7). Tigecycline, once considered a last-resort antibiotic, is now facing a global increase in resistance, with over 50% of multidrug-resistant *A. baumannii* demonstrating heterogeneous resistance to this drug (8).

Eravacycline, the world’s first fluorocycline antibacterial agent, is a promising alternative because of its broad-spectrum activity against common MDR bacteria (9). *In vitro* experiments have shown that the activity of eravacycline against *A. baumannii* is 8 times and 2 times greater than those of minocycline and tigecycline, respectively (10). Despite these *in vitro* findings, clinical evidence supporting the use of eravacycline in the treatment of cIAIs, particularly in the context of increasing tigecycline resistance, is lacking. This trial aims to fill this evidence gap by evaluating the clinical efficacy and safety of eravacycline compared with those of tigecycline in patients in the ICU with cIAIs.

## 2 Study design

**Figure 1:**
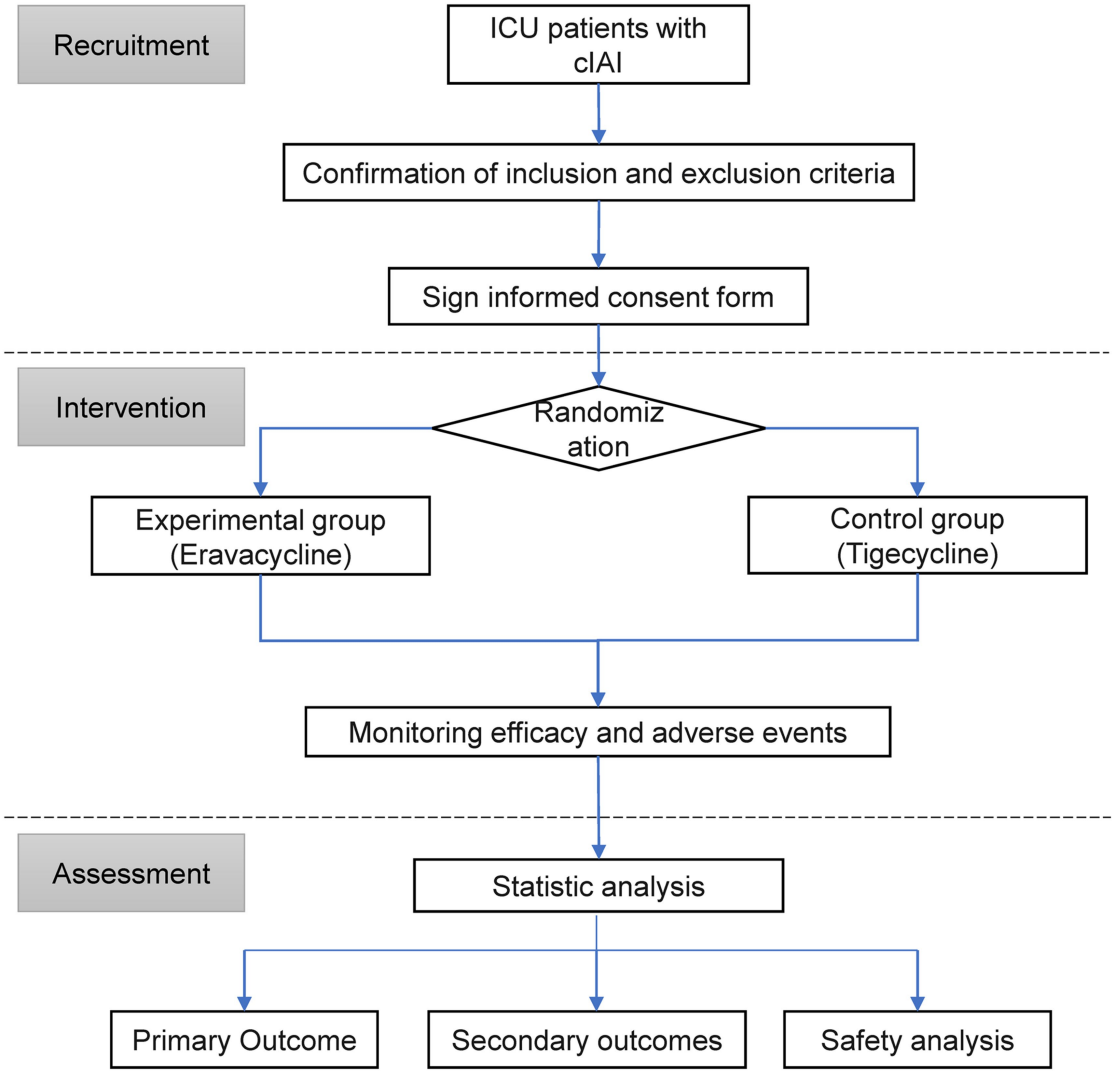
Flow chart. ICU, intensive care unit; cIAI, complicated intra-abdominal infection.

This study is a multicenter, randomized, single-blind, tigecycline-controlled study involving 5 centers/hospitals. The trial was fully approved by the Ethics Committee of Ansteel Group General Hospital [(2023) Ethics Review No. (14)] and was registered in the Chinese Clinical Trial Registry^1^ with the listed primary and secondary endpoints. This study will be conducted in accordance with the clinical trial protocol (and any revisions), the Declaration of Helsinki (current revision), the international guidelines for the management of intra-abdominal infections, and the Chinese expert consensus (11, 12). The research design is shown in Figure 1.

## 3 Research environment

The patients are registered and being treated in the ICUs of 5 centers/hospitals in China: (1) Ansteel Group General Hospital, (2) Dalian Municipal Central Hospital Affiliated with Dalian University of Technology, (3) Affiliated Zhongshan Hospital of Dalian University, (4) Jinzhou Municipal First People’s Hospital, and (5) Central Hospital of Zhuanghe City.

## 4 Patient selection

According to the guidelines, cIAI is defined as an infection that breaks through the primary organ affected and enters the abdominal cavity, causing peritonitis or an abdominal abscess (13). In accordance with the inclusion and exclusion criteria, eligible patients will be registered and randomly assigned to receive intravenous infusions of either eravacycline or tigecycline.

### 4.1 Inclusion criteria

1. ICU inpatients with cIAI; 2. Patients are 18 to 85 years old, without sex restrictions; 3. Patients are transferred to the ICU after surgery, with confirmation of an intra-abdominal infection (involving pus in the abdominal cavity) with peritonitis; 4. confirmation of infection through surgical intervention within 24 h after admission to the ICU: evidence of a systemic inflammatory response, consistent with physical examination results of intra-abdominal infection, and supportive imaging findings of intra-abdominal infection; 5. patients whose infected lesions have been effectively removed or drained or who do not require surgical intervention; and 6. the patient or their authorized person fully understands the purpose and significance of the trial, voluntarily participates and signs an informed consent form containing contact information.

### 4.2 Exclusion criteria

1. Patients who are known to have allergies to any excipients containing eravacycline, tigecycline, tetracycline, or investigational drug formulations;
2. Patients with intra-abdominal lesions that are unlikely to have infectious primary causes, such as inflammatory bowel disease, liver abscess, abdominal wall abscess, intestinal obstruction without perforation, or ischemic bowel without perforation;
3. Patients with intra-abdominal infections suspected to be caused by fungi, parasites, viruses, or pulmonary tuberculosis, except in cases of upper intestinal perforation where empirical coverage of fungi is often given, will not be excluded;
4. The exclusion criteria related to antibiotics are as follows: a. within 72 h prior to randomization, effective antimicrobial therapy was used for >24 h [however, patients with clear baseline pathogens and treatment failure after antibiotic treatment for at least 72 h may be enrolled]; b. cases in which cIAI is known to be caused by a pathogen resistant to one of the investigational drugs; c. patients who receive treatment for the current infection with investigational drugs or similar drugs; or d. in addition to research drugs, it is also necessary to use nonresearch antibacterial drugs in combination;
5. An anticipated need for systemic antibiotic treatments lasting more than 14 days;
6. Obvious liver disease, liver function damage (Child–Pugh C grade), or possible signs of liver disease and/or abnormal laboratory test results: a. ALT or AST > 5xULN; b. total bilirubin>3xULN;
7. Patients with congenital or acquired immunodeficiency diseases, those who have received organ transplantation within the previous year of enrollment, or those who have received immunosuppressive drug treatment within 30 days before enrollment;
8. Patients who are within 7 days prior to randomization or who are expected to use potent CYP3A inducers, such as phenytoin sodium, rifampicin, or carbamazepine, during the study period;
9. Patients with systemic malignant tumors that require chemotherapy, immunotherapy, radiation therapy, or antitumor treatment within the 3 months prior to total organic compound (TOC) visits;
10. Pregnant women, lactating women, or those with a fertility plan within 2 weeks after the end of the study;
11. Patients who have participated in clinical trials of other drugs or medical devices within 1 month prior to screening;
12. Patients who are expected to have a survival time or treatment intention of less than 6– to 8 weeks; and.
13. Patients who, as determined by the researchers, do not meet the criteria for inclusion in clinical trials for various reasons, such as concomitant brain herniation, acute myocardial infarction, pulmonary embolism, or ECMO treatment.

### 4.3 Criteria for early withdrawal from treatment

Early withdrawal from treatment refers to withdrawal from the study treatment before the completion of the treatment course. Patients who withdraw from treatment early are not considered withdrawn from the study early and should continue to complete the follow-up visit process. The study will employ an intention-to-treat analysis, which includes all patients who were initially randomized to receive the study drug, regardless of whether they completed the full course of treatment. This approach ensures that the analysis reflects the provided treatment options’ real-world effectiveness. The criteria for early withdrawal from treatment are as follows:

1. The patient or their authorized person requests to withdraw from treatment;
2. Patients whose conditions worsen or do not improve after 72 h of continuous use of the investigational drug and who have been determined by the researcher to no longer benefit from continued use of the investigational drug;
3. After the investigational drug is applied, if the sample culture strain is resistant to the drug, the clinical efficacy is evaluated by the researcher as persistent or progressive;
4. Individuals who experience intolerable adverse events/serious adverse events and for whom it has been determined by the researcher that continuing to use the investigational drug would pose a greater risk to the patient than its benefits;
5. Cases in which protocol violations occur, such as receiving other effective antimicrobial treatments (excluding those specified in the protocol) or other concomitant medications prohibited by this protocol during the study period, which, in the judgment of the researcher, might seriously affect the elimination, metabolism, or efficacy evaluation of the investigational drug;
6. Other situations that require early withdrawal from treatment.

### 4.4 Exit criteria

All patients who signed a written informed consent form and were screened as qualified to enter the study had the right to withdraw at any time. The exit criteria were as follows:

1. The patient or their authorized person refuses to continue the study or voluntarily withdraws informed consent;
2. Patient death;
3. Patients who are lost to follow-up;
4. Poor compliance, no longer accepting the medication or testing before completing all studies, or inability to adhere to the study protocol to complete the study;
5. Other situations that the researchers believe require withdrawal.

## 5 Test group

Patients who meet the inclusion criteria will be randomly assigned to the experimental group or control group at a 1:1 ratio. Tigecycline was chosen as a control drug because it is currently recommended by multiple authoritative guidelines/consensuses both domestically and internationally for the treatment of multidrug-resistant bacterial infections. In many countries, including the United States and China, tigecycline is commonly used in cases involving drug-resistant bacteria or other antibiotics and has poor efficacy for treating abdominal infections (11, 14, 15). Using a central stratified block randomization method, the subjects were randomly assigned to receive either eravacycline or tigecycline at a 1:1 ratio.

Although this study employs a single-blind design, we recognize the possibility of bias resulting from the clinicians’ awareness of the treatment allocation. To address this potential issue, we will include a blinded clinical assessor who will be unaware of the treatment assignments. The assessor will be responsible for conducting independent clinical evaluations and providing objective measurements in conjunction with the evaluations conducted by the investigators. This supplementary measure guarantees that a crucial element of our study remains free from bias, thereby enhancing the overall integrity of our findings. The remainder of the blinding procedure will adhere to the original protocol, ensuring that neither patients nor data analysis researchers are aware of the treatment groups, thus maintaining the integrity of the study.

When patients are registered after signing the consent form, detailed information about the anti-infective treatment, baseline demographics, and disease severity will be recorded before the start of the study.

## 6 Study drug management

Two research drugs (eravacycline and tigecycline) have been approved for marketing by the National Medical Products Administration in China. The approved indications include complex abdominal infections in adults. Therefore, this study strictly followed the approved dosage for intravenous administration according to the instructions. The recommended dose of eravacycline is 1 mg/kg, which is administered every 12 h, and the duration of intravenous infusion is approximately 60 min. The recommended dosage for tigecycline is 100 mg for the first dose, followed by 50 mg every 12 h for intravenous infusion lasting 30–60 min. All the study treatments will last for 4–14 days. The total duration of treatment will be determined by clinical doctors based on the severity of the infection, patient clinical response, imaging studies, progress in bacteriology, and when possible, consultation with surgical and infectious disease experts. For patients with renal dysfunction or those who are undergoing hemodialysis, the dosage of the investigational drug does not need to be adjusted. The dose adjustment for patients with liver function impairment is shown in Table 1.

**Table 1:** Dose adjustment of the study drug.

	Eravacycline	Tigecycline
Normal hepatic function	1 mg/kg q12h	50 mg q12h (First dose 100 mg)
Child–Pugh A	1 mg/kg q12h	50 mg q12h (First dose 100 mg)
Child–Pugh B	1 mg/kg q12h	50 mg q12h (First dose 100 mg)
Child–Pugh C	On the first day, 1 mg/kg every 12 h Starting the next day, 1 mg/kg every 24 h	25 mg q12h (First dose 100 mg)
Renal insufficiency	No adjustment required	No adjustment required
Continuous renal replacement therapy	No adjustment required	No adjustment required

Resistance to tigecycline will be routinely tested as part of the study protocol. In cases where resistance is identified, the study will manage crossover treatments based on predefined criteria, and data on eravacycline resistance will also be collected and tested routinely to guide clinical decisions and ensure patient safety.

If a patient shows clinical improvement after ≥4 days of intravenous study treatment, the treatment can be stopped. If *Pseudomonas aeruginosa* is suspected or isolated, open-label amikacin or gentamicin can be added to any protocol based on the researcher’s judgment. Antifungal therapy is prohibited unless there is clear evidence of a pathogen. The research plan aims to minimize the likelihood of previous use of other antibiotics, which may confound the evaluation of treatment efficacy.

During the administration process, circulatory and respiratory functions will be continually monitored, and airway assistance measures, artificial ventilation, and other resuscitation devices will be readily accessible. Symptoms and fluctuations before and after administration will be recorded in the patients’ and nurses’ records. When adverse reactions occur, the symptoms, medication duration, dosage, and intervention measures will be recorded.

Eravacycline was acquired from Everest Medicine (Singapore) Pte Ltd., specification 50 mg, batch number AT3072B; tigecycline was obtained from CTTQ PHARMA, specification 50 mg, batch number 230504115.

The protocol will include a comprehensive screening process for known CYP3A4 inducers and inhibitors (Appendix). Patients with potent CYP3A4 inducers will be excluded from the study based on exclusion criteria. Patients on moderate or weak CYP3A4 inducers will be monitored closely, but no dosage adjustment will be made unless deemed necessary by the treating physician. In addition, patients on CYP3A4 inhibitors will be evaluated for potential drug–drug interactions, and appropriate measures will be taken to minimize the risk of adverse effects.

The protocol prohibits using medications that may interfere with the study drug’s efficacy or safety profile. This includes antibiotics with similar mechanisms of action or known to affect the study outcomes. A comprehensive list of prohibited medications is included in the study protocol’s Appendix, which will be provided to all participating sites and made available to investigators and participants upon request (Appendix).

## 7 Population definition

1. The microbiologic modified intention to treat (mITT) population will include all patients with a confirmed diagnosis of complex abdominal infections (cIAIs) and positive infection source samples and/or blood sample cultures at baseline [excluding pathogens not expected to respond to either trial drug, such as nonfermenting gram-negative bacilli (e.g., *P. aeruginosa*, fungi, or mycobacteria)]. The mITT population will be used for demographic and baseline purposes.
2. Clinical evaluation (CE) population: In the mITT population, researchers will follow the requirements of important components of the study protocol [such as cultivating baseline pathogenic gram-negative pathogens (reported in infection source samples and/or blood samples)], excluding those patients with pathogens (*P. aeruginosa*) who do not respond as expected to the investigational drug. This population will be used for clinical efficacy and all-cause mortality analysis.
3. Microbiologically evaluable (ME) population: In the mITT population, individuals who have completed the study in accordance with important components of the study protocol and obtained baseline pathogenic gram-negative pathogens (reported in infection sources and/or blood samples) are expected to respond to the investigational drug. This population will be used for clinical efficacy, microbiological efficacy, comprehensive efficacy, and all-cause mortality analyses.
4. Safety analysis (SS) population: Patients who have received at least one dose of the investigational drug during the clinical study period. The safety analysis population will be used for the analysis of safety data.

## 8 Effectiveness evaluation

The primary efficacy end point is the all-cause 30-day mortality of patients in clinically evaluable and microbiologically evaluable populations.

The secondary efficacy endpoints are as follows: (1) the proportion of patients with a total response (composite endpoint consisting of clinical cure and microbiological response) in the clinically evaluable population at the end of treatment (days 5–14) and the test of cure visits (days 12 ± 2); (2) the proportion of total responsive patients in the microbiome-evaluable population at the end of treatment and the test of cure visits; (3) the proportion of microbiologically evaluable patients in the population with a total response to each pathogenic bacterium at the end of treatment and the test of cure visits; (4) the total response proportion of patients with drug-resistant bacterial infections in the microbiome-evaluable population at the end of treatment and the test of cure visits; (5) ICU hospitalization time and expenses; (6) quality of life outcomes or functioning after ICU discharge as assessed by application of the EuroQol-5D and Barthel Index scales; (7) changes in Sequential Organ Failure Assessment (SOFA) scores, reflecting the impact of the treatment on organ dysfunction over time; and (8) time to clinical improvement, measured from the start of treatment until the first observation of significant improvement in clinical status.

Given the variable susceptibility of MDR pathogens to tetracyclines, our study protocol will include analyses stratified by bacterial species. Specifically, subgroup analyses will be conducted for CRAB and CRE to gain a deeper understanding of the comparative efficacy of eravacycline versus tigecycline.

## 9 Safety evaluation

Safety will be assessed based on adverse events (ADRs) related to the investigational drug, changes in clinical laboratory test results (i.e., hematological screening, clinical biochemical tests, and urine analysis), electrocardiograms (ECGs), and vital signs during the trial period. Possible adverse event items are shown in Table 2.

**Table 2:** List of possible adverse events.

Systematic organ classification	Adverse events
General	Abdominal pain
	Abscess
	Weakness
	Headache
	Infect
Cardiovascular system	Phlebitis
	Thrombophlebitis
	QTc interval extension
Digestive system	Diarrhea
	Indigestion
	Nausea
	Vomit
	Acute pancreatitis
Blood and lymphatic system	Anemia
	APTT extension
	PT extension
	Thrombocytopenia
	INR elevation
	Hypofibrinogenemia
Metabolism and nutrition	AST elevation
	ALT elevation
	Bilirubin elevation
	Amylase elevation
	ALP elevation
	BUN elevation
	Poor wound healing
	Hypoglycemia
	Hyponatremia
	Hypoalbuminemia
Respiratory system	Pneumonia
Nervous system	Dizziness
Immune system	Allergic/Allergic like Reactions
Skin and accessory structures	Rash
	Pruritus
	Severe skin reactions, including Stevens Johnson syndrome

APTT, activated partial thromboplastin time; PT, prothrombin time; INR, international normalized ratio; AST, aspartate transaminase; ALT, alanine aminotransferase; ALP alkaline phosphatase; BUN, blood urea nitrogen.

Given the documented gastrointestinal toxicity associated with tigecycline, the study protocol will include comprehensive plans for monitoring and documenting specific gastrointestinal adverse events. The Gastrointestinal Symptom Rating Scale (GSRS), a validated tool for assessing gastrointestinal symptoms, will systematically record and rate the severity of gastrointestinal adverse events, including nausea, vomiting, diarrhea, and abdominal pain.

## 10 Data management and monitoring

All raw data (Table 3) will be meticulously recorded in the Case Report Form (CRF) by designated researchers. To ensure accuracy, each data entry will be independently double-checked by two individuals. The research coordinator at each center will supervise the data collection process, and the chief investigator, database administrator, and the statistician responsible for the statistical analysis will jointly determine the optimal database location strategy.

**Table 3:** List of data collection methods.

	Variables
Demographic data	Date of birth, sex, height, weight
Trial characteristics	Dates of screening and enrollment, inclusion criteria and consent details, date and time of randomization
Past medical history and risk factors	Past 3 months’ medical history, concomitant diseases, surgical history, allergic history, medication history (including immunosuppressive drugs, cytotoxic drugs, and biological agents), participation in other drug or medical device clinical trials
Infection and prognostic assessment	cIAI diagnosis time, presumed source of infection, invasive operation for source control, adequacy of source control, residue lesions, ICU admission time, APACHE II score, SOFA score
Antibiotic data	After the onset of the disease, all antibiotic usage, type/time/dose/route/frequency information
Clinical observations	Daily vital signs (blood pressure, heart rate, respiration, body temperature), clinical symptoms and signs, urine output, hematological variables, renal variables, hepatic variables, coagulation function, blood gas analysis, CRP, PCT, use of vasoactive drugs, catecholamine index, use of renal replacement therapy, mechanical ventilation duration, concomitant medication
Microbiological data	Date and time of initial peritoneal drainage culture, full susceptibility profile, daily peritoneal drainage culture results days 1 to 4; any further positive peritoneal drainage cultures and pathogen detection/resistance characteristics; other clinical sites growing *Escherichia*, *Klebsiella* or *Acinetobacter*, any multidrug-resistant organism, *P. aeruginosa* or *Clostridium difficile* identified within 30 days
Outcome data	Survival at 30 days post randomization Date of death or discharge Total response (composite endpoint consisting of clinical cure and microbiological response) at the end of treatment and the test of cure visits Length of ICU stay and costs Protocol violations and adverse events Reasons for trial withdrawal

cIAI, complicated intra-abdominal infection; ICU, intensive care unit; APACHE, Acute Physiology and Chronic Health Evaluation; SOFA, Sequential Organ Failure Assessment; CRP, C-reactive protein; PCT, procalcitonin.

An independent Data Safety and Monitoring Board (DSMB) will be constituted to further enhance the safety and integrity of the study. The DSMB, comprising experts with pertinent knowledge and experience, will operate independently of the study team. Its remit is to conduct regular reviews of the accumulating trial data, to ensure the safety of participants and the validity of the trial results. The DSMB will have the authority to recommend to the sponsor whether to continue, modify, or stop the trial, based on its data review insights.

## 11 Statistics

This study is a randomized controlled clinical trial. The two groups are the eravacycline group and the tigecycline group, with a ratio of 1:1. All-cause 30-day mortality is the main outcome measure. The researchers plan to use noninferiority tests for the ratio of two propositions to estimate the required sample size, assuming a noninferiority ratio of 2. Owing to the lack of randomized clinical trials in this specific field, sample size estimates were obtained from Xavier Guirao et al.’s observational study on the treatment of intra-abdominal infections with TGC (16). In their study, the all-cause mortality of patients treated with TGC (our control group) was 18.7%. Our previous results revealed that the mortality rate of the eravacycline group (17.7%) was slightly lower than that of the tigecycline group, with an estimated actual ratio of 0.95. The total sample size calculated through PASS (version 15.0.5) is N = 262 cases to perform a unilateral analysis *α*, achieving 80% efficacy at a level of 0.025. Considering a 10% dropout rate, the total number of subjects required for the last two groups is 292, with at least 146 subjects in each group. In light of the considerable variability observed in ICU mortality rates, an interim analysis will be conducted once 50% of the data has been collected. This analysis will facilitate a reassessment of the effect sizes and power of the study. If the initial mortality rates diverge significantly from the anticipated estimates, the sample size will be adjusted accordingly to ensure the integrity and statistical power of the study are maintained.

The intention to improve treatment will be analyzed based on microbiology, including all randomized subjects, who will be evaluated within their randomized groups. For continuous numerical variables, the quantity, mean, median, standard deviation, minimum, maximum, and coefficient of variation (CV, if applicable) will be determined after an independent sample t test or Wilcoxon rank sum test. The categorical variables will be reported as rates (percentages) and will be analyzed using the Pearson X^2^ test or Fisher’s exact probability method. The baseline will be defined as the last nonmissing observation data collected before the first use of the study drug. The Shapiro–Wilk test and QQ plot in SAS will be used to check the normality of the data. All the statistical analyses will be performed using SAS software (version 9.1 or higher; SAS Institute), and all the statistical inferences will be conducted using a two-sided test with a statistical significance level of 0.05. Confidence intervals (CIs) will be calculated and reported for all endpoints, thereby providing a comprehensive interpretation of the clinical relevance and statistical significance of the findings. Missing data will be classified as response uncertainty, and in the analysis of the microbiological intention-to-treat population, this will be considered treatment failure.

We will calculate and report CIs for all secondary endpoints, such as treatment response rates, to provide a comprehensive interpretation of the clinical relevance and statistical significance of our findings.

### 11.1 Analysis of the main effectiveness results

The purpose of this experiment is to evaluate the difference in treatment efficacy between the experimental group (eravacycline) and the control group (tigecycline). The statistical hypotheses are as follows: H_0_: P1/P2 ≥ R0 (the all-cause mortality rate of the experimental group is lower than or equal to that of the control group); H_1_: P1/P2 = R1 < R0 (the all-cause mortality rate in the experimental group is greater than that in the control group). The main efficacy indicator is the comparison of the all-cause mortality rate, which is a qualitative indicator and will be analyzed using the Pearson X^2^ test or Fisher’s exact probability method.

### 11.2 Analysis of secondary effectiveness results

The Pearson X^2^ test or Fisher’s exact probability method will be used to compare the differences between the two groups at different visit times and the proportion of total-response patients in different analysis populations. The length and cost of ICU hospitalization will be evaluated using independent sample t tests or Wilcoxon rank sum tests.

### 11.3 Safety analysis

Independent sample t tests will be used to evaluate changes in clinical laboratory test results, compare the incidence of adverse events between two groups using the Pearson X^2^ test, and provide a list of adverse events that occurred in this trial.

## 12 Discussion

As the world’s first and currently the only approved new broad-spectrum fluorocycline antibiotic, there are very few clinical trials related to eravacycline. To our knowledge, this is the first clinical trial to investigate the efficacy and safety of eravacycline in high-risk patients in the ICU with complex intra-abdominal infections, especially compared with those of classic tigecycline.

Tigecycline is a unique class of semisynthetic glycylcycline antibiotics used to treat various microbial infections caused by multidrug-resistant gram-positive and gram-negative pathogens (17). The intravenous administration of tigecycline has been approved for severe skin and soft tissue infections, complex intra-abdominal infections, and community-acquired bacterial pneumonia in adults. Owing to its broad antibacterial spectrum and strong bactericidal ability, it has become a powerful weapon in the clinical treatment of complex abdominal infections. However, with years of widespread use, the prevalence of TGC resistance has been increasing annually worldwide. Two recent studies have clearly indicated that mobile tigecycline resistance (MTR) has become an emerging health disaster that requires urgent global intervention (18, 19). Moreover, the United States Food and Drug Administration has issued a black box warning indicating that tigecycline can increase the mortality rate. Considering the high mortality rate of patients in the ICU with complex abdominal infections and the risk of infection with multidrug-resistant bacteria, ICU physicians are always vigilant during the medication process. Seeking effective and safe drugs for the treatment of complex abdominal infections is a constant problem that is continually being addressed by ICU physicians.

Compared with tigecycline, eravacycline is a fully synthetic fluorocycline antibacterial drug. Eravacycline has been specifically modified on the D-ring: fluorine atoms are used to replace the C7 position, and pyrrolidone acetamide groups are used to replace the C9 position, achieving stronger antibacterial activity, improving drug permeability, increasing the tissue concentration, and increasing metabolic stability (20, 21). Eravacycline has a wide antibacterial spectrum and can cover common gram-positive, gram-negative, anaerobic bacteria and atypical pathogens, except for *P. aeruginosa*, especially MDRs that produce serine and metalloenzymes. Furthermore, eravacycline is either not affected or rarely affected by common tetracycline efflux pumps (tetA, tetB, tetK) and ribosomal protective protein (tetM) resistance genes (22). In most of the strains tested in *in vitro* experiments, the MIC of eravacycline was 2–4 times lower than that of tigecycline (9). Therefore, unlike tigecycline, physicians can reduce concerns about multidrug-resistant bacteria. In addition, in a phase I clinical trial of healthy subjects, the plasma and lung exposure levels of eravacycline were significantly greater than those of tigecycline (9.12 vs. 3.70 μg·h/ml and 9.18 vs. 6.32 μg·h/ml, respectively) (23, 24), suggesting the potential for enhanced clinical efficacy compared with that of tigecycline.

Owing to its lower apparent distribution volume (321 L vs. 639 L) and significantly shorter half-life than tigecycline (20 h vs. 42.4 h), eravacycline may reduce the adverse effects of drug accumulation. In their respective phase III clinical trials, digestive system adverse reactions were the most common, with an incidence rate of eravacycline<omadacycline<tigecycline (22). Other possible adverse reactions can be observed in the central nervous system; metabolic function; and the mental, urinary, and respiratory systems. Overall, the incidence rates of adverse reactions with eravacycline are lower than those with tigecycline and omadacycline (25). Recent meta-analyses have shown that, compared with that of tigecycline, the number of drug discontinuations due to adverse events is significantly lower (OR = 0.17, 95% CrI = 0.03–0.81) (26).

In summary, these findings demonstrate the enormous potential of this drug as a novel candidate antibiotic in the ICU. If the drug exhibits the same beneficial characteristics as those in previous studies in patients with severe and complex intra-abdominal infections, it could become a new choice for patients and clinical physicians.

## Footnotes

^1^ChiCTR2300078646, www.chictr.org.cn/showproj.html?proj=208164
