# Supplementary material for: Efficacy and safety of eravacycline versus tigecycline for complicated intra-abdominal infections in the ICU: a multicenter, single-blind, parallel randomized controlled trial study protocol
Source: Front Med (Lausanne). 2024 Nov 22;11:1496402. doi: 10.3389/fmed.2024.1496402 (PMC11620859; doi:10.3389/fmed.2024.1496402)
Supplement: Supplementary file 1 [file Data_Sheet_1.DOCX]

Supplementary Material

# List of Known CYP3A4 Inducers and Inhibitors

## Strong CYP3A4 Inhibitors:

- Adagrasib

- Atazanavir

- Ceritinib

- Clarithromycin

- Cobicistat and cobicistat-containing coformulations

- Darunavir

- Idelalisib

- Indinavir

- Itraconazole

- Ketoconazole

- Levoketoconazole

- Lonafarnib

- Lopinavir

- Mifepristone (when used chronically)

- Nefazodone

- Nelfinavir

- Nirmatrelvir-ritonavir

- Ombitasvir-paritaprevir-ritonavir

- Ombitasvir-paritaprevir-ritonavir plus dasabuvir

- Posaconazole

- Ritonavir and ritonavir-containing coformulations

- Saquinavir

- Tucatinib

- Voriconazole

## Moderate CYP3A4 Inhibitors:

- Amiodarone

- Aprepitant

- Avacopan

- Berotralstat

- Cimetidine

- Conivaptan

- Crizotinib

- Cyclosporine

- Diltiazem

- Duvelisib

- Dronedarone

- Erythromycin

- Fedratinib

- Fluconazole

- Fosamprenavir

- Fosaprepitant

- Fosnetupitant-palonosetron

- Grapefruit juice

- Imatinib

- Isavuconazole (isavuconazonium sulfate)

- Lefamulin

- Letermovir

- Netupitant

- Nilotinib

- Nirogecestat

- Ribociclib

- Schisandra

- Verapamil

## Strong CYP3A4 Inducers:

- Carbamazepine

- Phenytoin

- Rifampin (rifampicin)

## Moderate CYP3A4 Inducers:

- Bexarotene

- Bosentan

- Cenobamate

- Dabrafenib

- Dexamethasone

- Dipyrone

- Efavirenz

- Elagolix, estradiol, and norethindrone

- Eslicarbazepine

- Etravirine

- Lorlatinib

- Mitapivat

- Modafinil

- Nafcillin

- Pexidartinib

- Repotrectinib

- Rifabutin

- Rifapentine

- Sotorasib

- St. John's wort

Note: This list may not be exhaustive and includes some of the most commonly recognised CYP3A4 inducers and inhibitors. It's important to note that the degree of inhibition or induction can vary based on dosage, method, and timing of administration.

# List of Prohibited Medications

Below is a list of medications prohibited for patients participating in this study. These medications are restricted because they may interfere with the efficacy or safety of the study drug or affect the accuracy of the study outcomes. This list will be provided to all investigators and study sites and can be made available to participants upon request.

1. Antibiotics

Any antibiotics with mechanisms of action similar to the study drug.

Other antibiotics known to impact study outcomes.

2. Drug Interactions

Medications known to significantly affect the pharmacokinetics or pharmacodynamics of the study drug.

Medications metabolized by CYP3A4 that may interact with the study drug. Strong CYP3A inducers (such as phenytoin sodium, rifampicin, carbamazepine, etc.).

3. Immunomodulators

Immunosuppressive drugs.

4. Antifungal Medications

Use of antifungal medications, other than those permitted in the study protocol, will be restricted.

5. Other Medications

Any other medications that may interfere with the interpretation of study results.

6. Complementary and Alternative Medicines

Any complementary and alternative medicines that may affect the course of infection or the effectiveness of treatment.

Note: This list is not exhaustive and may need to be adjusted based on the specific circumstances of the patient and the progress of the study. All prohibited medications must be discussed in detail at the time of obtaining informed consent from the patient and should be regularly updated and reviewed during the study period.

Investigators must ensure that patients do not use any medications that may affect the results of the study during the study period. For any questions or specific situations, investigators should consult the study protocol or contact the principal investigator for guidance.
